# Cytokines and Epidermal Lipid Abnormalities in Atopic Dermatitis: A Systematic Review

**DOI:** 10.3390/cells12242793

**Published:** 2023-12-08

**Authors:** Parth R. Upadhyay, Lucia Seminario-Vidal, Brian Abe, Cyrus Ghobadi, Jonathan T. Sims

**Affiliations:** Eli Lilly and Company, Lilly Corporate Center, Indianapolis, IN 46285, USAcyrus.ghobadi@gmail.com (C.G.); sims_jonathan_thomas@lilly.com (J.T.S.)

**Keywords:** cytokines, atopic dermatitis, lipids, skin barrier, barrier dysfunction, ceramides, fatty acid, cholesterol

## Abstract

Atopic dermatitis (AD) is the most common chronic inflammatory skin disease and presents a major public health problem worldwide. It is characterized by a recurrent and/or chronic course of inflammatory skin lesions with intense pruritus. Its pathophysiologic features include barrier dysfunction, aberrant immune cell infiltration, and alterations in the microbiome that are associated with genetic and environmental factors. There is a complex crosstalk between these components, which is primarily mediated by cytokines. Epidermal barrier dysfunction is the hallmark of AD and is caused by the disruption of proteins and lipids responsible for establishing the skin barrier. To better define the role of cytokines in stratum corneum lipid abnormalities related to AD, we conducted a systematic review of biomedical literature in PubMed from its inception to 5 September 2023. Consistent with the dominant T_H_2 skewness seen in AD, type 2 cytokines were featured prominently as possessing a central role in epidermal lipid alterations in AD skin. The cytokines associated with T_H_1 and T_H_17 were also identified to affect barrier lipids. Considering the broad cytokine dysregulation observed in AD pathophysiology, understanding the role of each of these in lipid abnormalities and barrier dysfunction will help in developing therapeutics to best achieve barrier homeostasis in AD patients.

## 1. Introduction

Atopic dermatitis (AD) is a complex and highly heterogeneous skin inflammatory disease that affects up to 20% of the world population [1]. Clinically, the disease is characterized by pruritus, lichenification, and xerosis [2]. Due to their recurrent and chronic course, these skin manifestations severely affect the quality of life and pose significant morbidity in affected patients [3,4]. In addition, there are also disease features that vary depending on age, race and ethnicity, disease severity, and geographical location [3,4].

The pathogenesis of AD is also complex; it encompasses an intricate interplay between a dysfunctional epidermal barrier, immune hyper-activation, and microbial dysbiosis. The “outside-in” hypothesis of AD suggests that a compromised epidermal barrier allows the penetration of external allergens, triggering the infiltration of immune cells to cause skin inflammation [5]. In contrast, the “inside-out” hypothesis describes inflammatory cytokines in the skin preceding and leading to barrier dysfunction [5]. While the primary mechanism that causes the disease is still debated, the epidermal barrier is the main target site of disease pathology and morbidity [6].

The epidermal barrier defects observed in AD are primarily observed in the stratum corneum and stratum granulosum. The stratum corneum consists of flattened, anucleate corneocytes densely packed with keratin fibers aggregated by filaggrin (FLG) and extracellular lipid lamellar matrix [7]. The corneocytes and extracellular lipid layers are tethered by a structure called the cornified envelope, which consists of crosslinked barrier proteins, including FLG, within corneocytes [8]. A loss-of-function mutation in *FLG* is the strongest genetic risk factor for AD. Reduced expression of FLG is associated with compromised skin barrier and loss of natural moisturizing factors [9]. In particular, reductions in triglycerides and ceramide species were significant in patients with *FLG* mutations [10]. Reduced FLG expression in AD has been proposed to cause cytoskeletal defects in the stratum corneum, which impairs lamellar body secretion, leading to the disorder of extracellular lipids [11]. The extracellular lipid lamellar matrix of the stratum corneum consists of ceramides (Cer), free fatty acids (FFAs), and cholesterol (CHOL). Ceramide lipids consist of more than 300 species belonging to 12 classes, which are defined by specific types of fatty acid (non-hydroxy [N], α-hydroxy fatty acid [A], and esterified ω-hydroxyl [EO]) and sphingoid bases (dihydrosphingosine [dS], sphingosine [S], phytosphingosine [P], and 6-hydroxysphingosine [H]) [12]. Fatty acids of varying lengths, ranging from 12 up to 36 carbon numbers, are present in the human stratum corneum, whereas the majority of cholesterol is synthesized in the stratum corneum in situ from acetyl-CoA [13]. Each of these three major types of lipids is present in the stratum corneum, with approximately 50% of the lipid mass contributed by ceramides, more than 25% cholesterol, 15% FFAs, and the remaining mass filled by phospholipids, which are different than membrane phospholipids [14]. These lipids are mainly formed in differentiated keratinocytes in the stratum granulosum layer, packed into lamellar bodies, and co-processed by lipid metabolizing enzymes such as lipid elongases, phospholipases, glucocerebrosidase (GCase), and acid sphingomyelinases (SMase) [14,15]. Table 1 summarizes the lipid metabolizing enzymes and their roles. The lipids are then secreted in the extracellular space of the stratum corneum and, in conjunction with corneocytes, form a “brick and mortar” structure of the stratum corneum. The resulting barrier prevents entry of environmental material into the skin layers and prevents trans-epidermal water loss (TEWL) and electrolyte losses from the skin [14]. Indeed, an inverse relationship between skin lipid content and TEWL indicates the cardinal role of epidermal lipids in epidermal barrier integrity.

There is unambiguous evidence that epidermal barrier disruption in AD exhibits altered composition and organization of lamellar lipids. Ceramide levels are significantly reduced in the stratum corneum of AD skin compared to healthy skin [16,17,18]. It has been reported that total ceramide levels and larger species of ceramides (>40 total carbons) like Cer[NH], Cer[EOS], Cer[NP], Cer[EOH], and Cer[EOP] are expressed at significantly lower levels, whereas smaller species (<40 total carbons) are expressed at higher levels in AD skin compared to healthy counterparts [19]. Indeed, the long ceramide chains are essential for the formation of a tightly packed lipid barrier, and the short ceramides render the skin more permeable [20]. Similarly, in healthy human stratum corneum FFA composition, approximately 60% are of greater length than 20 carbons, whereas 40% are of less than 20 carbons [21]. In AD skin, however, long FFAs (>20 carbons) are decreased, and short FFAs (<20 carbons) are significantly increased (both by approximately 50%) compared to healthy skin [22]. The resulting decrease in the average FFA chain length is also associated with short-chain ceramides [22].

In the last decade, studies have reported that epidermal barrier dysfunction in AD is associated with cytokine responses [23]. Cytokines expressed in specific compartments of the skin and skin layers possess the most relevance for AD [24,25]. Type 2 inflammation, which is a predominant hallmark of AD, causes an increase in cytokines that lead to epidermal barrier dysfunction through a decrease in levels of skin barrier proteins [26]. Particularly, interleukin-4 (IL-4) and IL-13 have been reported to cause significant downregulation of proteins involved in the formation of the cornified envelope (i.e., FLG, loricrin, and involucrin), and inhibition of IL-4 restores the expression levels of these important regulators, demonstrating the role of type 2 cytokines in regulating epidermal barrier function in AD [27,28,29]. However, the direct role of cytokines in regulating the above-mentioned barrier lipids in AD is less appreciated [23]. The purpose of this systemic literature review is to summarize the role of cytokines in regulating epidermal lipid metabolism in AD and suggest the development of a comprehensive molecular module focused on lipid metabolism for use in future studies in this field.

## 2. Methods

### 2.1. Eligibility Criteria and Evidence Search

This work followed the updated PRISMA 2020 guidelines for the systematic review. This review protocol was not registered in any public registry. Inclusion and exclusion criteria for studies of interest were pre-determined before conducting the literature search for this systemic review. The literature search included the effects of cytokines in skin lipids in AD. All clinical and non-clinical, in vitro, and in vivo studies were included to achieve maximum rigor in the data summarized herein. Included studies were limited to those published in English, but no other restrictions were imposed. The search was conducted on PubMed from its inception to 5 September 2023 to enable the inclusion of all peer-reviewed articles. The bibliographies of relevant articles were also searched for potentially eligible studies.

The Medline search strategy was as follows: ((“cytokin”[All Fields] OR “cytokine s”[All Fields] OR “cytokines”[MeSH Terms] OR “cytokines”[All Fields] OR “cytokine”[All Fields] OR “cytokinic”[All Fields] OR “cytokins”[All Fields]) AND (“lipid s”[All Fields] OR “lipidate”[All Fields] OR “apidates”[All Fields] OR “apidates”[All Fields] OR “lipidation”[All Fields] OR “lipidations”[All Fields] OR “lipide”[All Fields] OR “lipides”[All Fields] OR “lipidic”[All Fields] OR “lipids”[MeSH Terms] OR “lipids”[All Fields] OR “lipid”[All Fields]) AND (“dermatitis, atopic”[MeSH Terms] OR (“dermatitis”[All Fields] AND “atopic”[All Fields]) OR “atopic dermatitis”[All Fields] OR (“atopic”[All Fields] AND “dermatitis”[All Fields])) AND (“skin”[MeSH Terms] OR “skin”[All Fields]) AND (“barrier”[All Fields] OR “barrier s”[All Fields] OR “barriers”[All Fields])).

### 2.2. Selection of Studies

**Figure 1:**
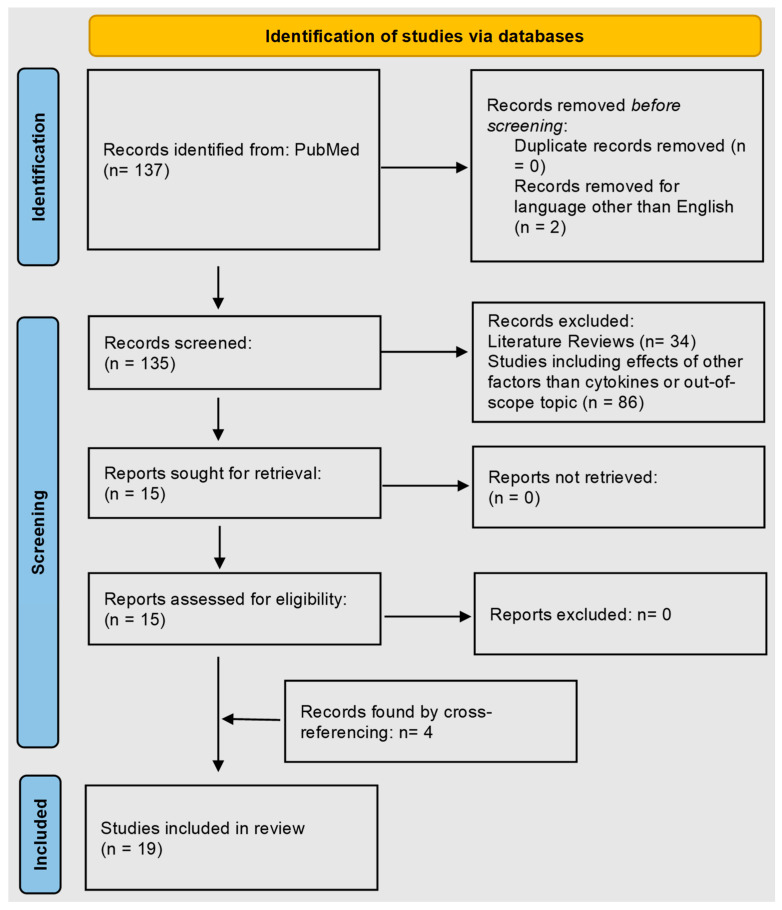
PRISMA flow diagram identifying studies on the role of cytokines in epidermal lipid abnormalities in atopic dermatitis.

The search query resulted in 137 non-duplicate results on PubMed. Out of the 137 articles gathered from searching PubMed, 2 articles were excluded based on language other than English. Two authors independently confirmed the search strategies and screened studies. The bias assessment was not performed using any automation tool. The competing reviews were resolved by making a consensus with all other authors. Further abstract screening led to the selection of 15 articles. The screening was based on choosing articles with a direct effect of cytokines on lipid metabolism in AD skin or showing a correlation of cytokines with lipid abnormalities. Additionally, 4 articles were included in this study, which were acquired from cross-references. The systemic search is depicted in the PRISMA diagram (Figure 1).

### 2.3. Data Extraction

As this review is descriptive, numeric data was not extracted for statistical analysis. Qualitative information regarding the associations of interest was extracted from the included publications.

## 3. Results

The 19 studies identified through this systematic review were published between 2005 and 2023. Table 2 summarizes the main characteristics of the studies included.

## 4. Discussion

### 4.1. Role of T_H_2-Associated Cytokines in Epidermal Lipid Abnormalities

T_H_2 cells are mainly responsible for the secretion of IL-4, IL-5, IL-13, and IL-31 cytokines in AD [5]. It is clear that the combination of T_H_2 cytokines IL-4, IL-13, and IL-31, when present in the skin, induces inflammation and AD-like phenotype [48,49]. Experiments on full-thickness human skin equivalents to determine whether skin inflammation affects epidermal lipid biosynthesis showed that treatment with a cocktail of IL-4, IL-13, and IL-31 resulted in significantly lower mRNA levels of genes encoding ELOVL1, acid SMase and β-GCase, which are involved in lipid chain elongation and ceramide synthesis, suggesting a direct role of T_H_2 cytokines on lipid biosynthesis and alterations in AD [33]. A meta-analysis-derived AD transcriptome profile bolstered the involvement of T_H_2 immune activation in the suppression of lipid metabolism-related genes, including *LPL*, *CES1*, *FA2H*, *ELOVL3*, and *FASN*, as shown by a strong inverse correlation [32]. Moreover, GCase activity and glucosyl-cholesterol levels strongly correlate with the proinflammatory cytokines and T_H_2 cytokines in the stratum corneum of pediatric AD [36]. Furthermore, the levels of ceramide markers sphinganine, Cer [S], Cer [dS], and GlcCER [S] in AD lesional skin were found to be inversely correlated with T_H_2 immune response and local cytokine milieu, suggesting the role of type 2 cytokines in abnormal metabolism of these lipids in AD skin [34].

Hatano et al. first reported that treatment of human epidermal sheets with IL-4 resulted in significant downregulation of glucocerebrosidase (GCase) and sphingomyelinase (SMase) mRNA, enzymes involved in ceramide production and reduced ceramide levels, which correlated with increased TEWL [38]. These results were further confirmed on acetone-treated living skin equivalents and human epidermal equivalents [30,39]. The transgenic mouse model of IL-13-driven AD exhibits profound abnormalities in stratum corneum lipids that are very similar to human AD at the molecular level. In this mouse model and in human keratinocyte cultures, IL-13 and IL-4 decreased the levels of lipid elongases ELOVL3 and ELOVL6 in lesional skin, suggesting their role In rendering epidermal lipids of short lengths in AD [40]. The reduction in acid sMase, gCase, lipid, and ceramide levels by IL-4 and IL-13 cytokines has been reported to be mediated by activation of the downstream signal transducer and activator of transcription 6 (STAT6) signaling pathway [31,37,40]. Furthermore, IL-4 and IL-13 cytokines were found to reduce lamellar body formation in differentiated keratinocytes [31]. These findings suggest that IL-4 and IL-13 cytokines mediate lipid abnormalities by downregulating enzymes involved in lipid synthesis and elongation and by decreasing lamellar bodies through STAT6 signaling.

The effects of IL-4 and IL-13 on epidermal lipids downstream of STAT signaling might be mediated through multiple mechanisms. A study on human sebaceous gland cells treated with IL-4 or IL-13 reported that activation of STAT6 by these cytokines leads to transcriptional upregulation of 3β-hydroxysteroid dehydrogenase 1 (HSD3B1), which results in an enhancement in androgen production that drives lipid abnormalities in sebocytes and keratinocytes. Particularly, IL-4 and IL-13 decreased the total amount of triglycerides in these cells, and this effect was abolished by siRNA targeting HSD3B1 [35]. Further, administration of the IL-4Rα blocker dupilumab results in downregulation of *HSD3B1* gene expression and subsequent lipid abnormalities in AD skin [35]. Consistent with this, treatment of AD human subjects with bi-weekly administration of dupilumab over 16 weeks normalized the levels of ceramides with non-hydroxy fatty acids and C18-sphingosine and the levels of esterified ω-hydroxy fatty-acid-containing ceramides [41]. Furthermore, it also increased the length of ceramides in the lesional and non-lesional skin of AD patients and significantly improved TEWL in the skin [41]. Additionally, in a placebo-controlled 16-week trial, it was found that dupilumab increased expression levels of the *ELOVL3* gene [29]. These findings solidify the involvement of IL-4/IL-13 cytokine signaling in epidermal barrier lipid abnormalities in AD skin through multiple mechanisms and suggest that patients receiving therapies targeting IL-4/IL-13 axis not only benefit by dampening the immune response but also by reversing the dysfunctional barrier through increasing epidermal lipid lengths, their total levels, and by increasing the expression of genes involved in forming the cornified envelope.

One of the hallmarks of AD skin is its *S. aureus* colonization, which is associated with skin barrier dysfunction. Investigating the mechanism of bacterial inhibition of skin barrier function, Kim et al. recently reported that *S. aureus* inhibits expression levels of ELOVL3 and ELOVL4 in human keratinocytes indirectly through IL-1β, TNF-α, IL-16, and IL-33, resulting in the reduction in very long fatty acid species [37]. Further, neutralization of these cytokines resulted in the restoration of *S. aureus*-induced inhibition of ELOVL3 [37]. This is particularly significant for methicillin-resistant *S. aureus* compared to methicillin-sensitive *S. aureus*, as the resistant version of bacterial colonization caused more prominent induction of these cytokines and inhibition of fatty acid elongases, resulting in lipid abnormalities [37]. Furthermore, T_H_2-cytokine-mediated decreases in SMase exacerbate *S. aureus*-induced keratinocyte death and lipid abnormalities through the effect of α-toxin [31]. Importantly, the prevalence of methicillin-resistant *S. aureus* in AD skin is increasing, and its colonization is associated with disease severity [37]. These data indicate that T_H_2 and other cytokines are necessary for deleterious effects mediated by *S. aureus* on epidermal lipids, and blocking these cytokines may result in the reversal of bacterial induction of lipid abnormalities.

In atopic dermatitis, IL-31 is mainly known to induce pruritus through neuro-immune interaction. However, this cytokine may have a role in epidermal barrier function [50]. In the Leiden epidermal model, IL-31 treatment decreased the relative abundance of ω-hydroxy ceramides and induced spongiosis [45]. In the three-dimensional organotypic skin model with either primary keratinocytes or HaCaT cells expressing inducible IL-31 receptor, IL-31 treatment significantly reduced overall lipid content and ceramide levels in the cornified envelope [43]. In this study, mRNA expression levels of lipid metabolism enzymes SMase, SMS, STS, and PLA2 were not affected by IL-31 treatment, and the authors proposed that IL-31 signaling might affect lipid metabolism at the post-translational level [43]. These enzymes have indeed been previously reported to be regulated by different post-translational controls [51,52,53]. However, in response to IL-31, their post-translational regulation and activity levels remain to be delineated. A study utilizing the N/TERT-based epidermal model, however, found that IL-31 treatment resulted in significant downregulation of mRNAs encoding SCD1 and GCase, enzymes that are involved in FFA and ceramide synthesis, respectively [44]. The precise mechanism and molecular pathway by which IL-31 regulates the expression of the lipid metabolism enzymes or levels of barrier lipids is yet to be determined. Further, IL-31-induced pruritus may trigger itch–scratch cycle, which can damage the stratum corneum and lipid distribution. The correlation between pruritus and barrier lipid abnormalities needs to be established.

In addition to its effects on epidermal lipids, the T_H_2 cytokines IL-4, IL-13, and IL-31, as well as the alarmins, thymic stromal lymphopoietin (TSLP), and IL-33, reduce FLG expression levels and thereby contribute to a compromised epithelial barrier [27,43,54,55,56]. Furthermore, T_H_2 cytokines also downregulate the expression of loricrin and involucrin, proteins that are involved in the formation of a cornified envelope and downregulated in AD skin [28,57]. These observations suggest that the inhibitory effects of T_H_2-dominant cytokines on stratum corneum lipid composition, lipid lengths, and lamellar body formation are in line with their inhibitory effects on barrier proteins, resulting in a dysfunctional epidermal barrier.

### 4.2. Role of T_H_17-Associated Cytokines in Epidermal Lipid Abnormalities

T_H_17 cells have been implicated in the pathogenesis of AD, particularly in patients of Asian ancestry and in patients with severe disease [58,59]. Studies indicate that IL-17 contributes to skin inflammation by increasing keratinocyte production of granulocyte–macrophage colony-stimulating factor (GM-CSF), TNF-α, IL-8, and vascular endothelial growth factor (VEGF) [60,61].

In vitro studies suggest that IL-17 affects the epidermal barrier by reducing the expression of FLG, IVL, and LOR genes with demonstrated involvement in functional barrier formation [62,63,64]. Linking to this, a single in vivo study was identified where both oxazolone and TPA-induced AD mouse models using IL-17^−/−^ and WT Balb/c mice suggested that IL-17 contributes to abnormal distribution of lamellar bodies in the skin, accompanied by edema, and TEWL [42]. Interestingly, T_H_2 cytokines were found to be decreased in the IL-17^−/−^ mice compared to their wild-type counterparts, irrespective of oxazolone or TPA exposure [42]. Consistent with this, IL-17A has been reported to induce T_H_2 signaling in another murine model of AD [65], and IL-17C has been shown to induce AD-like lesions in the MC903 mouse model [66]. Considering the activation of T_H_2 signaling, augmentation of keratinocyte-derived cytokines, and following skin inflammation, it is plausible that these AD-like characteristics and lipid changes induced by IL-17 occur as a secondary mechanism through activation of type 2 inflammation. Whether these IL-17-associated lipid abnormalities and epidermal lesions in the skin are attributed to the direct effect of IL-17 or via activation of T_H_2 response in AD is not known yet and warrants investigation.

### 4.3. Role of T_H_1-Associated Cytokines in Epidermal Lipid Abnormalities

T_H_1 activation is usually seen in patients with chronic AD [67]. Particularly in patients with the intrinsic endotype, where normal serum levels of IgE are detected, T_H_1 responses have been reported to be stronger than in patients with the extrinsic phenotype, where elevated serum IgE levels are seen [68]. T_H_1 cells mainly produce IFN-γ, TNF-α, and GM-CSF, which are detected in the chronic phase of the disease [67,69]. In the human epidermal keratinization model, treatment with TNF-α resulted in slight augmentation of ceramide levels, with an increase in serine-palmitoyl transferase-1/2, acid SMase, and β-GCase transcription levels [30]. The protein levels or enzyme activity in response to TNF-α were not studied. However, in the multilayer keratinocyte-based Leiden epidermal model system, TNF-α treatment led to decreased levels of saturated fatty acids with greater than 20 carbons, reduction in cholesterol levels, and induction of spongiosis [45]. These differences in the effects of TNF-α could be due to differences in the epidermal differentiation, lipid synthesis, and distribution in these model systems and disparate endpoints studied, which might be specific to the ceramide synthesis and/or lipid chain elongation processes.

The treatment of an epidermal keratinization skin equivalent model with IFN-γ was reported to significantly increase the gene expression levels of serine-palmitoyl transferase-1/2, β-GCase, acid SMase, and acid ceramidase. However, protein expression levels and enzymatic activity were not consistent with the changes in gene expression levels, which were supported by a non-significant increase in ceramide levels in the stratum corneum [30]. Analyses using a multilayer keratinocyte-based epidermal construct and mite fecal-antigen-induced AD-like dermatitis in an NC/Nga mouse model reported that IFN-γ decreases the mRNA levels of ELOVL1, ELOVL4, ELOVL6, and ELOVL7, and ceramide synthase, enzymes that are involved in elongation of fatty acid chains of ceramides and ceramide synthesis, and diminished levels of long-chain ceramides and fatty acids. These effects were found to be independent of STAT signaling [46,47]. While in the chronic phase of the disease, both T_H_1 and T_H_2 polarization is frequent, studies in AD patients with a positive T_H_1 profile, in addition to the presence of type 2 inflammation, are needed to further understand the role and function of IFN-γ.

Another T_H_1 cytokine, GM-CSF, has been reported to increase in AD skin and is mainly produced by T-cells and keratinocytes [70,71]. The production of GM-CSF may contribute to inflammatory response by activating immune cells like macrophages and dendritic cells in AD skin [72]. The role of GM-CSF on stratum corneum lipids was studied in the epidermal keratinization model, which found an increase in stratum corneum ceramide/total protein levels (μg/mg) and stimulation of acid SMase protein levels and enzyme activity followed by GM-CSF treatment [30]. Whether GM-CSF increases lamellar body formation, extrusion, and release remains to be understood. Further, the underlying molecular mechanism also needs to be unveiled.

Collectively, the role of T_H_1 cytokines in AD skin barrier remains ambiguous, as studies reported variable effects of TNF-α and IFN-γ on epidermal lipid metabolism and their levels. Future studies utilizing live human skin explants, in vivo AD models, and models employing multiple cytokine signatures as seen in AD are needed to unequivocally determine the role of T_H_1 cytokines and their mechanism of action on stratum corneum lipid metabolism and barrier function in AD patients.

### 4.4. Lipid Restoration Strategies in Atopic Dermatitis

Topical application of lipid-based formulations has been used as a strategy to restore barrier function in mild-to-moderate AD. Topical application of ceramide-, fatty-acid-, and cholesterol-based formulations restores the barrier structure and function and improves TEWL, pruritus, disease severity, and overall AD-associated discomfort levels [73,74,75,76].

The efficacy of lipid-based barrier restoration strategies supports the outside-in hypothesis of AD pathophysiology, where blocking penetration of external allergens improves AD-related symptoms. This suggests that topical application of lipid-based formulations should decrease skin inflammation and cytokine milieu. Indeed, in mouse models of AD, topical application of a ceramide derivative significantly reduced the skin expression levels of IL-4 and TNF-α [77,78]. Furthermore, in MC903, a vitamin D derivative, and ovalbumin models of AD, the application of linoleic acid-ceramide-rich topical emollient attenuated skin lesions and inflammation, which was accompanied by a decrease in serum levels of IL-4, TSLP, and IgE [79]. In patients with AD, ceramide- and magnesium-containing emollients significantly decreased stratum corneum levels of IL-4 and IL-13 [80,81]. These studies confirm that topical application of lipid-based formulations may help dampen the local and systemic cytokine levels. Studies with larger sample sizes in patients with diverse disease severities of AD are required for in-depth analysis of cytokine profiles associated with AD following lipid-based topical applications and to absolutely determine if topical lipid restoration strategies improve the AD-related cytokine and inflammatory profile at systemic levels.

Our systematic review results suggest that signaling mediated by type 2 cytokines and the JAK-STAT pathway are involved in lipid dysregulation in AD skin. In moderate-to-severe disease, administration of topical or systemic therapies targeting JAK inhibitors and IL-4R antagonists has shown significant improvements in barrier function with restoration of stratum corneum lipid homeostasis [29,41,82]. Further, IL-13-neutralizing antibody tralokinumab, which is approved in Europe for the treatment of AD, was reported to shift the stratum corneum lipid profile from lesional to non-lesional type, suggesting the underlying role in improving barrier function [83]. These data corroborate the inside-out hypothesis of AD, where dampening of hyperimmune activation results in restoration of barrier function. The IL-22 antibody fezakinumab and JAK inhibitors abrocitinib, upadacitinib, and baricitinib have also shown clinical efficacy in improving moderate-to-severe AD [67,84]. Whether IL-22 causes lipid abnormalities in AD skin and if fezakinumab and JAK inhibitors reverse barrier dysfunction and improve epidermal lipids warrant further investigation.

Further, in maintaining a healthy epidermal barrier, both protein and lipid components are crucial. In AD skin, barrier lipids, as well as protein levels, are decreased, as both FLG and lipid barrier genes are downregulated. However, whether FLG deficiency in AD is associated with abnormal epidermal lipids is ambiguous [85,86], and the co-dependency of barrier lipids and proteins needs to be investigated.

## 5. Challenges and Implications for Practice

As new information on the regulation of epidermal lipids in AD becomes available, implications with respect to pathogenesis and therapeutic approaches will be further clarified. For example, as noted above, the link between increases in circulating and local IL-4 and IL-13 with decreased and/or abnormal epidermal barrier lipids has been extensively studied. Since these observations, novel therapeutics targeting IL-4/IL-13 signaling have been approved for their use in AD or are under investigation, such as dupilumab, tralokinumab, and lebrikizumab. Clinical studies have confirmed that their administration restores epidermal lipid homeostasis in patients with moderate to severe AD [41,83].

As numerous cytokines are dysregulated in AD, a first step would be to test their combined effect on epidermal lipids in human skin equivalents to determine their respective contribution to epidermal lipid metabolism by testing the expression levels of lipid metabolism enzymes and levels of above-described lipid species. The ultimate aim would be to identify the cytokine(s) that need(s) to be targeted to provide the most effective epidermal barrier restoration in AD.

Less is known about the therapeutic effect of lipid-based topical formulations on skin inflammation in patients with AD since studies have been small, patients were not severely affected, and circulating cytokines were not quantified.

Further, epidermal barrier lipids in AD skin have been proposed for their use as predictive biomarkers. CER [DS], CER [S], and phytosphingosine, along with their ratio with other ceramide species, were found to be altered in infants who later developed AD [87]. A study utilizing tape strips from the skin of newborns to study stratum corneum lipid content reported that protein-bound ceramide levels were decreased, whereas short-chain non-hydroxy fatty acid sphingosine and alpha-hydroxy fatty acid sphingosine ceramides were elevated in children who later developed AD compared to healthy counterparts [88]. Thus, epidermal lipids, alone or in combination with a cytokine profile, could also be used as predictive biomarkers of AD.

## 6. Conclusions

**Figure 2:**
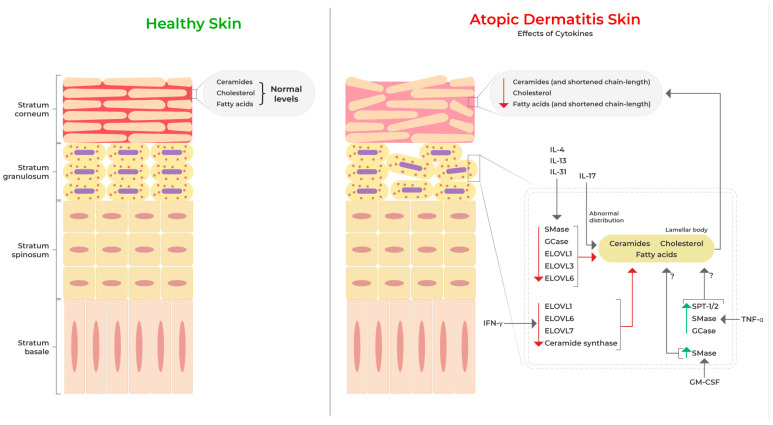
Effects of cytokines on epidermal barrier lipids in atopic dermatitis skin. Red arrows depict decreasing levels/effects, and green arrows indicate increasing levels. ? indicates uncertain effects.

The purpose of this systemic review was to delineate the role played by cytokines in epidermal lipid alterations, including their levels, metabolism, and barrier function in AD, and identify key molecular signatures that should be studied in greater detail in attempts to therapeutically restore barrier function. We found that studies so far have mainly focused on T_H_2-, T_H_1-, and T_H_17-related cytokines in determining their roles in AD skin barrier lipids. In AD lesions, the compromised barrier is linked with decreased levels of ceramides, fatty acids, and cholesterol in the disrupted stratum corneum. The T_H_2-skewed immune response in AD, along with T_H_1/T_H_17/T_H_22 polarization, contribute to the exacerbation of barrier dysfunction (Figure 2). Studies have clearly established that T_H_2 cytokines potently diminish the synthesis of long-chain ceramides and fatty acids and disrupt the distribution of lamellar bodies in the stratum corneum. Furthermore, these effects are reported to be mediated by STAT signaling activated by these cytokines in epidermal keratinocytes. In addition, therapies to dampen T_H_2 response also reinstate lipid homeostasis and barrier function, suggesting the cardinal role of these cytokines in mediating lipid defects in AD skin. Further, T_H_17-related cytokines also alter lipid lamellar structures in the skin and change T_H_2 cytokine profiles. The roles of T_H_22 cytokines on barrier lipids in AD, however, have yet to be elucidated. Further studies are required to understand the role of cytokines beyond T_H_2 and whether therapies targeting these cytokines might be beneficial in improving epidermal homeostasis and AD symptoms. The net cutaneous phenotype reflects a mixture of pathogenic and compensatory mechanisms in AD, and the presence of various T_H_-cell-derived cytokines reflects the complexity of AD lesions. Therefore, it is equally important to understand the mechanism of each cytokine in AD skin.

This systemic review found studies involving cytokines IL-4, IL-13, IL-31, IL-17, IL-31, IFN-γ, TNF-α, and GM-CSF for their effects on epidermal lipids in AD skin. In addition, it highlights the lack of studies investigating the effect on epidermal lipids of additional cytokines dysregulated in AD, such as TSLP, IL-22, IL-25, IL-33, IL-9, and IL-37 [25,49]. Most of the studies focused on cytokine analysis in AD skin have employed bulk or single-cell RNA sequencing, tissue-level proteomics, immunohistochemistry, ELISA, and Western blotting and have utilized skin biopsies or tape strips. These approaches provide global information on the present cytokines in the AD skin and potentially associated cell populations. Further, studies have investigated the role of specific cytokines in skin inflammation based on their involvement in the disease. However, owing to the complex inflammatory microenvironment in AD skin and pleiotropy of cell types in secreting cytokines, investigating the most relevant cytokines associated with barrier dysfunction and the effects of these cytokines in a spatial context within AD skin is extremely important. It is most likely that cytokines present in the surrounding environment have the most potent effect on barrier lipid metabolism. The processing and incorporation of ceramides, fatty acids, and cholesterol in the lamellar bodies mainly begins in the stratum granulosum layer of the epidermis [89]. FLG processing also begins in the same granular layer to form the stratum corneum layer [11]. Therefore, investigating the local cytokine milieu, specifically surrounding granular keratinocytes, in the context of enzymes responsible for lipid metabolism will enhance our understanding of cytokine action on the epidermal barrier. In addition to the approaches utilized to study the cytokine mentioned above, high dimensional multiplexed immunofluorescence and spatial transcriptomics should prove to be excellent tools to obtain these data. These approaches will advance knowledge of cytokine-driven epidermal barrier lipid abnormalities, barrier dysfunction in AD skin, and the development of cytokine-specific therapeutics that repair the barrier and manage inflammation to better treat AD and AD-related symptoms.

## Figures and Tables

**Table 1:** Lipid metabolism enzymes described in this article and their function.

Enzyme Name	Acronym	Function
Serine palmitoyltransferase 1/2	SPT-1/2	3-ketodihydrosphingosine synthesis; involved in de novo synthesis of ceramides
Beta-Glucocerebrosidase	β-GCase	Involved in the salvage pathway of ceramide synthesis; catalyzes glycosylceramide to ceramides
Sphingomyelinase	SMase	Hydrolyze sphingomyelin to produce ceramides
Sphingomyelin synthase	SMS	Synthesis of sphingomyelin
Ceramidase	CDase	Hydrolyze ceramide to form free sphingosine bases and fatty acids
Elongation of very-long-chain fatty acids 1/3/4/6/7	ELOVL 1/3/4/6/7	Elongation of saturated and unsaturated fatty acids
Stearoyl-CoA desaturase 1	SCD1	Biosynthesis of monounsaturated fatty acids
Fatty acid synthase	FASN	De novo synthesis of fatty acids
Fatty acid 2-hydroxylase	FA2H	Hydroxylation of fatty acids and production of 2-hydroxysphingolipids
Lipoprotein lipase	LPL	Generation of free fatty acids
3β-hydroxysteroid dehydrogenase 1	HSD3B1	Production of androgens
Steroid sulfatase	STS	Hydrolysis of aryl and alkyl steroid sulfates
Phospholipase A2 (cytosolic)	PLA2	Hydrolysis of fatty acids from membrane phospholipids

**Table 2:** Summary of studies on the role of cytokines in epidermal barrier lipid metabolism in this systemic review.

Study	Model System	Cytokine(s) Studied	Major Findings
Sawada et al. [30]	Human epidermal equivalents	↑ IL-4, ↑ IL-6	Decreased total ceramide; decreased expression of SPT-2, β-Gcase, Smase
		↑ TNF-α	Increased total ceramide, increased expression of SPT-1/2, β-Gcase, Smase
		↑ IFN-γ	No significant increase in ceramide; increased expression of SPT-1/2, β-Gcase, Smase, Cdase
		↑ GM-CSF	Increased ceramide; no change in gene expression
Brauweile et al. [31]	Primary human keratinocytes	↑ IL-4/↑ IL13	Reduced ceramide levels, lamellar body formation, and decreased expression of Smase; these effects are mediated by STAT6
Ewald et al. [32]	Meta-analysis of transcriptome	↑ T_H_2 cytokines	Inverse correlation of T_H_2 immune activation to the expression of lipid metabolism genes
Danso et al. [33]	Human skin equivalents	↑ IL-4, ↑ IL-13, and ↑ IL-31	Decreased expression of ELVOL1, acid Smase, and β-Gcase.
Toncic et al. [34]	Tape strips from human AD skin	↑ T_H_2 cytokines	Inverse correlation of T_H_2 cytokine milieu to ceramide levels and sphingoid bases.
Zhang et al. [35]	Human sebocytes, keratinocytes, and MC903 AD mouse model	↑ IL-4, ↑ IL-13	Decreased levels of fatty acids/triglycerides through STAT6-HSD3B1-mediated androgen production
Kezic et al. [36]	Tape strips from human AD skin	↑ T_H_2 cytokines, ↑ IL-18,↑ IL-1α	Correlation with Gcase activity and glucosylcholesterol levels.
Kim et al. [37]	Primary human keratinocytes	↑ IL-1β, ↑ TNF-α, ↑ IL-16, ↑ IL-33	Decreased ELOVL3 and ELOVL4 expression. The cytokine effects are associated with methicillin-resistant *S. aureus*
Hatano et al. [38]	Normal human keratinocytes and human epidermal sheets	↑ IL-4	Decreased Gcase expression; decrease in TNF-α and IFN-γ-induced Smase expression and ceramide levels
Hatano et al. [39]	Acetone-wounded living epidermis	↑ IL-4	Decrease in Smase, Gcase, and total ceramide levels
Berdyshev et al. [40]	Human keratinocytes, IL-13 transgenic mouse	↑ IL-4, ↑ IL-13	Increase in short-chain ceramides and decrease in long-chain ceramides; decreased ELOVL3/ELOVL6 expression in STAT6-dependent mechanism
Guttman-Yassky et al. [29]	Human AD Patients	↑ IL-4/↑ IL-13	Dupilumab (IL-4R antibody) increased expression of ELOVL3.
Berdyshev et al. [41]	Human AD patients	↑ IL-4/↑ IL-13	Dupilumab increased ceramide chain length and normalized lipid composition
Heo et al. [42]	Oxazolone induced-AD in IL-17^−/−^ Balb/c mice	↓ IL-17	Improvement in the distribution of lamellar bodies and lipid distribution
Cornelissen et al. [43]	3D organotypic skin equivalents	↑ IL-31	Decreased ceramide and lipid envelope; no effects on Smase, sphingomyelin synthase, steroid sulfatase, and phospholipase A2
Van Drongelen et al. [44]	N/TERT-based epidermal models	↑ IL-31	Decreased expression of stearoyl-CoA desaturase 1 and Gcase; no effect on Smase
Danso et al. [45]	Leiden epidermal model	↑ TNF-α	Decreased long-chain fatty acids, Cer [EO],
		↑ IL-31	Decreased ω-hydroxy ceramide
Tawada et al. [46]	3D epidermis	↑ IFN-γ	Decreased levels of long-chain ceramides; downregulation of ELOVL1/6/7 and ceramide synthase
Kanoh et al. [47]	Mite fecal antigen-induced AD-like dermatitis in NC/Nga	↑ IFN-γ	Decreased levels of long-chain ceramides; downregulation of ELOVL1 and ELOVL4

Abbreviations: IL-4, interleukin 4; IL-6, interleukin 6; IL13, interleukin 13; IL-31, interleukin 31; IL-16, interleukin 16; IL-1α, interleukin-1 alpha; IL-1β, interleukin-1 beta; IL-33, interleukin 33; IL-17, interleukin 17; TNF-α, tumor necrosis factor alpha; IFN-γ, interferon gamma; GM-CSF, granulocyte–macrophage colony-stimulating factor; SPT-1/2, serine palmitoyltransferase ½; β-Gcase, beta-glucocerebrosidase; Smase, sphingomyelinase; Cdase, ceramidase; STAT6, downstream signal transducer and activator of transcription 6; ELVOL1, elongation of very-long-chain fatty acids 1; ELVOL3, elongation of very-long-chain fatty acids 3; ELVOL6, elongation of very-long-chain fatty acids 6; IL-4R, interleukin-4 receptor; ELVOL4, elongation of very-long-chain fatty acids 4; HSD3B1, 3-beta-hydroxysteroid dehydrogenase 1. ↑ indicates an increase in cytokines in experimental conditions. ↓ indicates a decrease in cytokines in experimental conditions.

## Data Availability

Not applicable.

## References

[1] Kaufman B.P., Guttman-Yassky E., Alexis A.F. (2018). Atopic dermatitis in diverse racial and ethnic groups-Variations in epidemiology, genetics, clinical presentation and treatment. Exp. Dermatol..

[2] Yew Y.W., Thyssen J.P., Silverberg J.I. (2019). A systematic review and meta-analysis of the regional and age-related differences in atopic dermatitis clinical characteristics. J. Am. Acad. Dermatol..

[3] Silverberg J.I., Gelfand J.M., Margolis D.J., Boguniewicz M., Fonacier L., Grayson M.H., Simpson E.L., Ong P.Y., Chiesa Fuxench Z.C. (2018). Patient burden and quality of life in atopic dermatitis in US adults: A population-based cross-sectional study. Ann. Allergy Asthma Immunol..

[4] Drucker A.M., Wang A.R., Li W.Q., Sevetson E., Block J.K., Qureshi A.A. (2017). The Burden of Atopic Dermatitis: Summary of a Report for the National Eczema Association. J. Investig. Dermatol..

[5] Facheris P., Jeffery J., Del Duca E., Guttman-Yassky E. (2023). The translational revolution in atopic dermatitis: The paradigm shift from pathogenesis to treatment. Cell Mol. Immunol..

[6] Silverberg N.B., Silverberg J.I. (2015). Inside out or outside in: Does atopic dermatitis disrupt barrier function or does disruption of barrier function trigger atopic dermatitis?. Cutis.

[7] Proksch E., Brandner J.M., Jensen J.M. (2008). The skin: An indispensable barrier. Exp. Dermatol..

[8] Candi E., Schmidt R., Melino G. (2005). The cornified envelope: A model of cell death in the skin. Nat. Rev. Mol. Cell Biol..

[9] Stander S. (2021). Atopic Dermatitis. N. Engl. J. Med..

[10] Angelova-Fischer I., Mannheimer A.C., Hinder A., Ruether A., Franke A., Neubert R.H., Fischer T.W., Zillikens D. (2011). Distinct barrier integrity phenotypes in filaggrin-related atopic eczema following sequential tape stripping and lipid profiling. Exp. Dermatol..

[11] Elias P.M. (2014). Lipid abnormalities and lipid-based repair strategies in atopic dermatitis. Biochim. Biophys. Acta.

[12] Masukawa Y., Narita H., Shimizu E., Kondo N., Sugai Y., Oba T., Homma R., Ishikawa J., Takagi Y., Kitahara T. (2008). Characterization of overall ceramide species in human stratum corneum. J. Lipid Res..

[13] Norlen L., Nicander I., Lundsjo A., Cronholm T., Forslind B. (1998). A new HPLC-based method for the quantitative analysis of inner stratum corneum lipids with special reference to the free fatty acid fraction. Arch. Dermatol. Res..

[14] Feingold K.R., Elias P.M. (2014). Role of lipids in the formation and maintenance of the cutaneous permeability barrier. Biochim. Biophys. Acta.

[15] Elias P.M. (1983). Epidermal lipids, barrier function, and desquamation. J. Investig. Dermatol..

[16] Melnik B., Hollmann J., Hofmann U., Yuh M.S., Plewig G. (1990). Lipid composition of outer stratum corneum and nails in atopic and control subjects. Arch. Dermatol. Res..

[17] Imokawa G., Abe A., Jin K., Higaki Y., Kawashima M., Hidano A. (1991). Decreased level of ceramides in stratum corneum of atopic dermatitis: An etiologic factor in atopic dry skin?. J. Investig. Dermatol..

[18] Yamamoto A., Serizawa S., Ito M., Sato Y. (1991). Stratum corneum lipid abnormalities in atopic dermatitis. Arch. Dermatol. Res..

[19] Ishikawa J., Narita H., Kondo N., Hotta M., Takagi Y., Masukawa Y., Kitahara T., Takema Y., Koyano S., Yamazaki S. (2010). Changes in the ceramide profile of atopic dermatitis patients. J. Investig. Dermatol..

[20] Skolova B., Janusova B., Zbytovska J., Gooris G., Bouwstra J., Slepicka P., Berka P., Roh J., Palat K., Hrabalek A. (2013). Ceramides in the skin lipid membranes: Length matters. Langmuir.

[21] Ansari M.N., Nicolaides N., Fu H.C. (1970). Fatty acid composition of the living layer and stratum corneum lipids of human sole skin epidermis. Lipids.

[22] van Smeden J., Janssens M., Kaye E.C., Caspers P.J., Lavrijsen A.P., Vreeken R.J., Bouwstra J.A. (2014). The importance of free fatty acid chain length for the skin barrier function in atopic eczema patients. Exp. Dermatol..

[23] Brunner P.M., Israel A., Zhang N., Leonard A., Wen H.C., Huynh T., Tran G., Lyon S., Rodriguez G., Immaneni S. (2018). Early-onset pediatric atopic dermatitis is characterized by T(H)2/T(H)17/T(H)22-centered inflammation and lipid alterations. J. Allergy Clin. Immunol..

[24] Humeau M., Boniface K., Bodet C. (2022). Cytokine-Mediated Crosstalk Between Keratinocytes and T Cells in Atopic Dermatitis. Front. Immunol..

[25] Clausen M.L., Kezic S., Olesen C.M., Agner T. (2020). Cytokine concentration across the stratum corneum in atopic dermatitis and healthy controls. Sci. Rep..

[26] Beck L.A., Cork M.J., Amagai M., De Benedetto A., Kabashima K., Hamilton J.D., Rossi A.B. (2022). Type 2 Inflammation Contributes to Skin Barrier Dysfunction in Atopic Dermatitis. JID Innov..

[27] Howell M.D., Kim B.E., Gao P., Grant A.V., Boguniewicz M., Debenedetto A., Schneider L., Beck L.A., Barnes K.C., Leung D.Y. (2007). Cytokine modulation of atopic dermatitis filaggrin skin expression. J. Allergy Clin. Immunol..

[28] Kim B.E., Leung D.Y., Boguniewicz M., Howell M.D. (2008). Loricrin and involucrin expression is down-regulated by Th2 cytokines through STAT-6. Clin. Immunol..

[29] Guttman-Yassky E., Bissonnette R., Ungar B., Suarez-Farinas M., Ardeleanu M., Esaki H., Suprun M., Estrada Y., Xu H., Peng X. (2019). Dupilumab progressively improves systemic and cutaneous abnormalities in patients with atopic dermatitis. J. Allergy Clin. Immunol..

[30] Sawada E., Yoshida N., Sugiura A., Imokawa G. (2012). Th1 cytokines accentuate but Th2 cytokines attenuate ceramide production in the stratum corneum of human epidermal equivalents: An implication for the disrupted barrier mechanism in atopic dermatitis. J. Dermatol. Sci..

[31] Brauweiler A.M., Goleva E., Leung D.Y.M. (2014). Th2 cytokines increase Staphylococcus aureus alpha toxin-induced keratinocyte death through the signal transducer and activator of transcription 6 (STAT6). J. Investig. Dermatol..

[32] Ewald D.A., Malajian D., Krueger J.G., Workman C.T., Wang T., Tian S., Litman T., Guttman-Yassky E., Suarez-Farinas M. (2015). Meta-analysis derived atopic dermatitis (MADAD) transcriptome defines a robust AD signature highlighting the involvement of atherosclerosis and lipid metabolism pathways. BMC Med. Genom..

[33] Danso M., Boiten W., van Drongelen V., Gmelig Meijling K., Gooris G., El Ghalbzouri A., Absalah S., Vreeken R., Kezic S., van Smeden J. (2017). Altered expression of epidermal lipid bio-synthesis enzymes in atopic dermatitis skin is accompanied by changes in stratum corneum lipid composition. J. Dermatol. Sci..

[34] Toncic R.J., Jakasa I., Hadzavdic S.L., Goorden S.M., Vlugt K.J.G., Stet F.S., Balic A., Petkovic M., Pavicic B., Zuzul K. (2020). Altered Levels of Sphingosine, Sphinganine and Their Ceramides in Atopic Dermatitis Are Related to Skin Barrier Function, Disease Severity and Local Cytokine Milieu. Int. J. Mol. Sci..

[35] Zhang C., Chinnappan M., Prestwood C.A., Edwards M., Artami M., Thompson B.M., Eckert K.M., Vale G., Zouboulis C.C., McDonald J.G. (2021). Interleukins 4 and 13 drive lipid abnormalities in skin cells through regulation of sex steroid hormone synthesis. Proc. Natl. Acad. Sci. USA.

[36] Kezic S., McAleer M.A., Jakasa I., Goorden S.M.I., der Vlugt K.G., Beers-Stet F.S., Meijer J., Roelofsen J., Nieman M.M., van Kuilenburg A.B.P. (2022). Children with atopic dermatitis show increased activity of beta-glucocerebrosidase and stratum corneum levels of glucosylcholesterol that are strongly related to the local cytokine milieu. Br. J. Dermatol..

[37] Kim J., Kim B.E., Berdyshev E., Bronova I., Bin L., Bae J., Kim S., Kim H.Y., Lee U.H., Kim M.S. (2023). Staphylococcus aureus causes aberrant epidermal lipid composition and skin barrier dysfunction. Allergy.

[38] Hatano Y., Terashi H., Arakawa S., Katagiri K. (2005). Interleukin-4 suppresses the enhancement of ceramide synthesis and cutaneous permeability barrier functions induced by tumor necrosis factor-alpha and interferon-gamma in human epidermis. J. Investig. Dermatol..

[39] Hatano Y., Katagiri K., Arakawa S., Fujiwara S. (2007). Interleukin-4 depresses levels of transcripts for acid-sphingomyelinase and glucocerebrosidase and the amount of ceramide in acetone-wounded epidermis, as demonstrated in a living skin equivalent. J. Dermatol. Sci..

[40] Berdyshev E., Goleva E., Bronova I., Dyjack N., Rios C., Jung J., Taylor P., Jeong M., Hall C.F., Richers B.N. (2018). Lipid abnormalities in atopic skin are driven by type 2 cytokines. JCI Insight.

[41] Berdyshev E., Goleva E., Bissonnette R., Bronova I., Bronoff A.S., Richers B.N., Garcia S., Ramirez-Gama M., Taylor P., Praestgaard A. (2022). Dupilumab significantly improves skin barrier function in patients with moderate-to-severe atopic dermatitis. Allergy.

[42] Heo W.I., Lee K.E., Hong J.Y., Kim M.N., Oh M.S., Kim Y.S., Kim K.W., Kim K.E., Sohn M.H. (2015). The role of interleukin-17 in mouse models of atopic dermatitis and contact dermatitis. Clin. Exp. Dermatol..

[43] Cornelissen C., Marquardt Y., Czaja K., Wenzel J., Frank J., Luscher-Firzlaff J., Luscher B., Baron J.M. (2012). IL-31 regulates differentiation and filaggrin expression in human organotypic skin models. J. Allergy Clin. Immunol..

[44] van Drongelen V., Haisma E.M., Out-Luiting J.J., Nibbering P.H., El Ghalbzouri A. (2014). Reduced filaggrin expression is accompanied by increased Staphylococcus aureus colonization of epidermal skin models. Clin. Exp. Allergy.

[45] Danso M.O., van Drongelen V., Mulder A., van Esch J., Scott H., van Smeden J., El Ghalbzouri A., Bouwstra J.A. (2014). TNF-alpha and Th2 cytokines induce atopic dermatitis-like features on epidermal differentiation proteins and stratum corneum lipids in human skin equivalents. J. Investig. Dermatol..

[46] Tawada C., Kanoh H., Nakamura M., Mizutani Y., Fujisawa T., Banno Y., Seishima M. (2014). Interferon-gamma decreases ceramides with long-chain fatty acids: Possible involvement in atopic dermatitis and psoriasis. J. Investig. Dermatol..

[47] Kanoh H., Ishitsuka A., Fujine E., Matsuhaba S., Nakamura M., Ito H., Inagaki N., Banno Y., Seishima M. (2019). IFN-gamma Reduces Epidermal Barrier Function by Affecting Fatty Acid Composition of Ceramide in a Mouse Atopic Dermatitis Model. J. Immunol. Res..

[48] Hayden P.J., Petrali J.P., Stolper G., Hamilton T.A., Jackson G.R., Wertz P.W., Ito S., Smith W.J., Klausner M. (2009). Microvesicating effects of sulfur mustard on an in vitro human skin model. Toxicol. In Vitro.

[49] Zhou J., Gemperline D.C., Turner M.J., Oldach J., Molignano J., Sims J.T., Stayrook K.R. (2021). Transcriptomic Analysis of Healthy and Atopic Dermatitis Samples Reveals the Role of IL-37 in Human Skin. Immunohorizons.

[50] Singh B., Jegga A.G., Shanmukhappa K.S., Edukulla R., Khurana Hershey G.H., Medvedovic M., Dillon S.R., Madala S.K. (2016). IL-31-Driven Skin Remodeling Involves Epidermal Cell Proliferation and Thickening That Lead to Impaired Skin-Barrier Function. PLoS ONE.

[51] Cordella-Miele E., Miele L., Mukherjee A.B. (1990). A novel transglutaminase-mediated post-translational modification of phospholipase A2 dramatically increases its catalytic activity. J. Biol. Chem..

[52] Reagan J.W., Hubbert M.L., Shelness G.S. (2000). Posttranslational regulation of acid sphingomyelinase in niemann-pick type C1 fibroblasts and free cholesterol-enriched chinese hamster ovary cells. J. Biol. Chem..

[53] Tani M., Kuge O. (2009). Sphingomyelin synthase 2 is palmitoylated at the COOH-terminal tail, which is involved in its localization in plasma membranes. Biochem. Biophys. Res. Commun..

[54] Kim J.H., Bae H.C., Ko N.Y., Lee S.H., Jeong S.H., Lee H., Ryu W.I., Kye Y.C., Son S.W. (2015). Thymic stromal lymphopoietin downregulates filaggrin expression by signal transducer and activator of transcription 3 (STAT3) and extracellular signal-regulated kinase (ERK) phosphorylation in keratinocytes. J. Allergy Clin. Immunol..

[55] Sehra S., Yao Y., Howell M.D., Nguyen E.T., Kansas G.S., Leung D.Y., Travers J.B., Kaplan M.H. (2010). IL-4 regulates skin homeostasis and the predisposition toward allergic skin inflammation. J. Immunol..

[56] Seltmann J., Roesner L.M., von Hesler F.W., Wittmann M., Werfel T. (2015). IL-33 impacts on the skin barrier by downregulating the expression of filaggrin. J. Allergy Clin. Immunol..

[57] Guttman-Yassky E., Suarez-Farinas M., Chiricozzi A., Nograles K.E., Shemer A., Fuentes-Duculan J., Cardinale I., Lin P., Bergman R., Bowcock A.M. (2009). Broad defects in epidermal cornification in atopic dermatitis identified through genomic analysis. J. Allergy Clin. Immunol..

[58] Brunner P.M., Guttman-Yassky E. (2019). Racial differences in atopic dermatitis. Ann. Allergy Asthma Immunol..

[59] Koga C., Kabashima K., Shiraishi N., Kobayashi M., Tokura Y. (2008). Possible pathogenic role of Th17 cells for atopic dermatitis. J. Investig. Dermatol..

[60] Sugaya M. (2020). The Role of Th17-Related Cytokines in Atopic Dermatitis. Int. J. Mol. Sci..

[61] Krzysiek J., Lesiak A., Szybka M., Michalak A., Pastuszak-Lewandoska D., Grzegorczyk J., Ciazynska M., Narbutt J. (2022). The role of heterodimer IL-17-A/F in atopic dermatitis. Postepy Dermatol. Alergol..

[62] Gutowska-Owsiak D., Schaupp A.L., Salimi M., Selvakumar T.A., McPherson T., Taylor S., Ogg G.S. (2012). IL-17 downregulates filaggrin and affects keratinocyte expression of genes associated with cellular adhesion. Exp. Dermatol..

[63] Furue M. (2020). Regulation of Filaggrin, Loricrin, and Involucrin by IL-4, IL-13, IL-17A, IL-22, AHR, and NRF2: Pathogenic Implications in Atopic Dermatitis. Int. J. Mol. Sci..

[64] Tan Q., Yang H., Liu E., Wang H. (2017). P38/ERK MAPK signaling pathways are involved in the regulation of filaggrin and involucrin by IL-17. Mol. Med. Rep..

[65] Nakajima S., Kitoh A., Egawa G., Natsuaki Y., Nakamizo S., Moniaga C.S., Otsuka A., Honda T., Hanakawa S., Amano W. (2014). IL-17A as an inducer for Th2 immune responses in murine atopic dermatitis models. J. Investig. Dermatol..

[66] Vandeghinste N., Klattig J., Jagerschmidt C., Lavazais S., Marsais F., Haas J.D., Auberval M., Lauffer F., Moran T., Ongenaert M. (2018). Neutralization of IL-17C Reduces Skin Inflammation in Mouse Models of Psoriasis and Atopic Dermatitis. J. Investig. Dermatol..

[67] Weidinger S., Beck L.A., Bieber T., Kabashima K., Irvine A.D. (2018). Atopic dermatitis. Nat. Rev. Dis. Primers.

[68] Kabashima-Kubo R., Nakamura M., Sakabe J., Sugita K., Hino R., Mori T., Kobayashi M., Bito T., Kabashima K., Ogasawara K. (2012). A group of atopic dermatitis without IgE elevation or barrier impairment shows a high Th1 frequency: Possible immunological state of the intrinsic type. J. Dermatol. Sci..

[69] Tuzlak S., Dejean A.S., Iannacone M., Quintana F.J., Waisman A., Ginhoux F., Korn T., Becher B. (2021). Repositioning T(H) cell polarization from single cytokines to complex help. Nat. Immunol..

[70] Berker M., Frank L.J., Gessner A.L., Grassl N., Holtermann A.V., Hoppner S., Kraef C., Leclaire M.D., Maier P., Messerer D.A. (2017). Allergies—A T cells perspective in the era beyond the T(H)1/T(H)2 paradigm. Clin. Immunol..

[71] Pastore S., Fanales-Belasio E., Albanesi C., Chinni L.M., Giannetti A., Girolomoni G. (1997). Granulocyte macrophage colony-stimulating factor is overproduced by keratinocytes in atopic dermatitis. Implications for sustained dendritic cell activation in the skin. J. Clin. Investig..

[72] Esche C., de Benedetto A., Beck L.A. (2004). Keratinocytes in atopic dermatitis: Inflammatory signals. Curr. Allergy Asthma Rep..

[73] Chamlin S.L., Kao J., Frieden I.J., Sheu M.Y., Fowler A.J., Fluhr J.W., Williams M.L., Elias P.M. (2002). Ceramide-dominant barrier repair lipids alleviate childhood atopic dermatitis: Changes in barrier function provide a sensitive indicator of disease activity. J. Am. Acad. Dermatol..

[74] Na J.I., Hwang J.S., Park H.J., Kim D.H., Park W.S., Youn S.W., Huh C.H., Park K.C. (2010). A new moisturizer containing physiologic lipid granules alleviates atopic dermatitis. J. Dermatol. Treat..

[75] Kircik L.H., Del Rosso J.Q., Aversa D. (2011). Evaluating Clinical Use of a Ceramide-dominant, Physiologic Lipid-based Topical Emulsion for Atopic Dermatitis. J. Clin. Aesthet. Dermatol..

[76] Berardesca E., Barbareschi M., Veraldi S., Pimpinelli N. (2001). Evaluation of efficacy of a skin lipid mixture in patients with irritant contact dermatitis, allergic contact dermatitis or atopic dermatitis: A multicenter study. Contact Dermat..

[77] Kang J.S., Youm J.K., Jeong S.K., Park B.D., Yoon W.K., Han M.H., Lee H., Han S.B., Lee K., Park S.K. (2007). Topical application of a novel ceramide derivative, K6PC-9, inhibits dust mite extract-induced atopic dermatitis-like skin lesions in NC/Nga mice. Int. Immunopharmacol..

[78] Kang J.S., Yoon W.K., Youm J.K., Jeong S.K., Park B.D., Han M.H., Lee H., Moon E.Y., Han S.B., Lee C.W. (2008). Inhibition of atopic dermatitis-like skin lesions by topical application of a novel ceramide derivative, K6PC-9p, in NC/Nga mice. Exp. Dermatol..

[79] Zhang J., Xu X., Wang X., Zhang L., Hu M., Le Y., Chen L., Zheng J. (2023). Topical emollient prevents the development of atopic dermatitis and atopic march in mice. Exp. Dermatol..

[80] Koppes S.A., Brans R., Ljubojevic Hadzavdic S., Frings-Dresen M.H., Rustemeyer T., Kezic S. (2016). Stratum Corneum Tape Stripping: Monitoring of Inflammatory Mediators in Atopic Dermatitis Patients Using Topical Therapy. Int. Arch. Allergy Immunol..

[81] Park K.Y., Kim D.H., Jeong M.S., Li K., Seo S.J. (2010). Changes of antimicrobial peptides and transepidermal water loss after topical application of tacrolimus and ceramide-dominant emollient in patients with atopic dermatitis. J. Korean Med. Sci..

[82] He H., Guttman-Yassky E. (2019). JAK Inhibitors for Atopic Dermatitis: An Update. Am. J. Clin. Dermatol..

[83] ANNEX I—Summary of Product Characteristics. https://www.ema.europa.eu/en/documents/product-information/adtralza-epar-product-information_en.pdf.

[84] Guttman-Yassky E., Brunner P.M., Neumann A.U., Khattri S., Pavel A.B., Malik K., Singer G.K., Baum D., Gilleaudeau P., Sullivan-Whalen M. (2018). Efficacy and safety of fezakinumab (an IL-22 monoclonal antibody) in adults with moderate-to-severe atopic dermatitis inadequately controlled by conventional treatments: A randomized, double-blind, phase 2a trial. J. Am. Acad. Dermatol..

[85] Vavrova K., Henkes D., Struver K., Sochorova M., Skolova B., Witting M.Y., Friess W., Schreml S., Meier R.J., Schafer-Korting M. (2014). Filaggrin deficiency leads to impaired lipid profile and altered acidification pathways in a 3D skin construct. J. Investig. Dermatol..

[86] van Drongelen V., Alloul-Ramdhani M., Danso M.O., Mieremet A., Mulder A., van Smeden J., Bouwstra J.A., El Ghalbzouri A. (2013). Knock-down of filaggrin does not affect lipid organization and composition in stratum corneum of reconstructed human skin equivalents. Exp. Dermatol..

[87] Rinnov M.R., Halling A.S., Gerner T., Ravn N.H., Knudgaard M.H., Trautner S., Goorden S.M.I., Ghauharali-van der Vlugt K.J.M., Stet F.S., Skov L. (2023). Skin biomarkers predict development of atopic dermatitis in infancy. Allergy.

[88] Berdyshev E., Kim J., Kim B.E., Goleva E., Lyubchenko T., Bronova I., Bronoff A.S., Xiao O., Kim J., Kim S. (2023). Stratum corneum lipid and cytokine biomarkers at age 2 months predict the future onset of atopic dermatitis. J. Allergy Clin. Immunol..

[89] Bhattacharya N., Sato W.J., Kelly A., Ganguli-Indra G., Indra A.K. (2019). Epidermal Lipids: Key Mediators of Atopic Dermatitis Pathogenesis. Trends Mol. Med..

